# Modeling Alien Invasions: Plasticity May Hold the Key to Prevention

**DOI:** 10.1371/journal.pbio.0040411

**Published:** 2006-10-31

**Authors:** Liza Gross

The fossil record shows that plant and animal extinctions have always been part of life. But today, species are disappearing at an unprecedented rate, unable to keep pace with habitat loss and alien species invasions. Exotic invasive species can quickly displace indigenous species and disrupt ecological relationships that evolved over millions of years. Invasions often alter food sources or introduce novel competitors or predators, requiring that a species modify corresponding traits (related to physiology, life history, or behavior, for example) to survive in the transfigured landscape.

In a new study, Scott Peacor, Mercedes Pascual, and colleagues derive a theory to probe the factors underlying a successful invasion. Their model included three basic elements: competition between two species, a variable environment, and a “plastic” trait that undergoes adaptive changes in response to the shifting environment. The authors hypothesized that when a flexible, adaptive response to environmental variation (called phenotypic plasticity) increases fitness, it should enhance a species’ ability to invade and displace other species, once established. This fitness-related plasticity may explain why some exotic species become invasive and others don’t. As expected, phenotypic plasticity exerted a “profound effect” on alien invasions, with plastic species successfully invading or resisting against invasion by an inflexible opponent. But plasticity, the authors were surprised to discover, also dramatically reduced invasion when exhibited by both invader and resident, suggesting that phenotypic plasticity can affect invasion in an unforeseen manner, independently of the fitness advantage it provides over species without plasticity.

Peacor et al. modeled the invasion of a hypothetical food chain—with a predator, resident consumer, and food source—by an invading consumer. The model assumes random environmental fluctuations and different evolutionary histories for resident and invasive species, placing the invader at the disadvantage in a foreign environment. And though higher foraging effort affords higher reproductive potential, it also risks higher predation, for both resident and alien consumers (echoing real-life risks between energy gain and death). Adding or removing the predator provides the environmental variation, and variable predation risk induces a behavioral response in prey. Both types of consumers could either discern the presence or absence of a predator and evolve bimodal foraging behavior (the plastic phenotype) or were unresponsive and evolved one optimal behavior for both circumstances (the nonplastic phenotype). Invasion success was measured as the time to displace the resident consumer. When the model was run sans invasive species, plastic consumers almost always ate in the absence of predators and almost never in their presence. Nonplastic consumers, in contrast, evolved an intermediate strategy in which the probability of eating was the same (about 45%) in the presence or absence of a predator. When both resident and invader were nonplastic and had no competitive advantage (that is, the same probability of death), the invader replaced the resident. And when only the resident or invader had plasticity-enhanced fitness, the plastic resident successfully repelled the inflexible invader, and the plastic invader displaced the inflexible resident. But to the authors’ surprise, invasion was rapid when both consumers were nonplastic—yet did not occur when both consumers were plastic; plasticity effectively acted as a barrier to invasion unless invaders were given a huge competitive advantage (a 40% lower chance of death).

**Figure:**
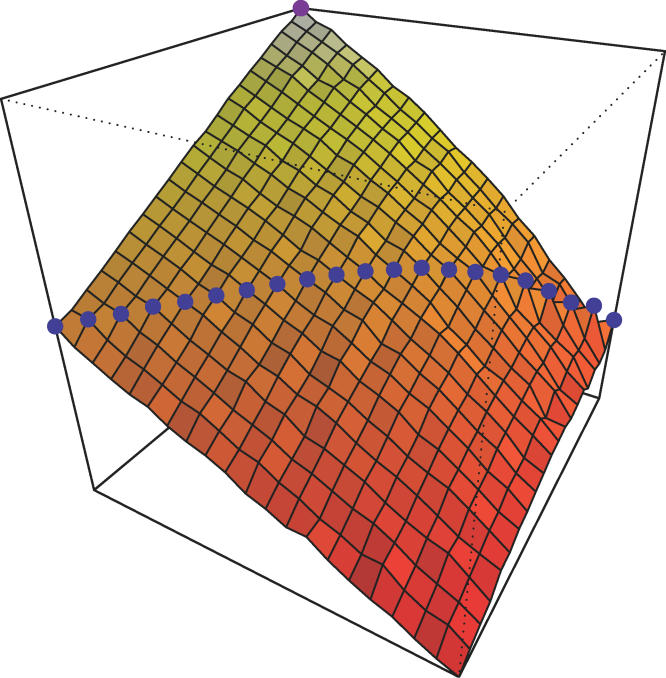
Phenotypic plasticity can increase the steepness of the fitness surface, which magni? es competitive differences and thereby acts as a barrier to species invasion.

To understand this puzzling pattern, the authors constructed a “fitness surface,” a graph plotting fitness as a function of the consumer’s foraging strategy (the probability of eating in the presence or absence of the predator). Peaks on this fitness landscape correspond to adaptive traits that increase fitness and valleys to those that decrease it. Plastic and nonplastic (whether resident or invading) consumers evolved optimal behavioral strategies that corresponded to quite different fitness surfaces—the graphs reflected their respective either/or (represented by a steep slope) and “average” (plateau, then decline) optimum foraging behaviors.

Since invaders had not undergone selection in the new environment, deviating from their foraging optimum could place them at a competitive fitness disadvantage. When both consumers were nonplastic, the alien incurred only minor fitness costs by deviating from the optimum, allowing it to eventually gain a foothold. But when both consumers had plasticity, the resident’s fitness landscape proved too steep to scale: when the invader strayed from its optimal strategy, it could no longer compete with the native, and died before reproducing—aborting the invasive process.

This model suggests that plasticity exerts a major influence on invasion by magnifying how even small differences in traits affect fitness. It also sheds light on natural invasive processes like colonization and vegetative succession—when new plant communities sequentially repopulate a landscape following fire, avalanche, or other disturbance—explaining how a vital community can spring from the ruins. The results also have implications for understanding species survival in fragmented landscapes, in which metapopulations persist by invading new habitat patches even as they go extinct in others.

